# Diatomite Dynamic Membrane Fouling Behaviour during Dewatering of *Chlorella pyrenoidosa* in Aquaculture Wastewater

**DOI:** 10.3390/membranes11120945

**Published:** 2021-11-29

**Authors:** Weiwei Huang, Weiguang Lv, Huaqiang Chu, Weiwei Lv, Wenzong Zhou, Bingzhi Dong

**Affiliations:** 1 Eco-Environment Protection Research Institute, Shanghai Academy of Agricultural Sciences, Shanghai 201403, China; hwwswx@163.com (W.H.); daqiyishi@163.com (W.L.); mengtang289@gmail.com (W.L.); 2 School of Environmental Science and Engineering, Tongji University, Shanghai 200092, China; hwwswx@126.com (H.C.); wzhhww@aliyun.com (B.D.); 3 Shanghai Runzhuang Agricultural Technology Co., Ltd., Shanghai 201403, China

**Keywords:** diatomite dynamic membrane, copper, *C. pyrenoidosa*, EOM, fouling

## Abstract

Combined microalgal and membrane filtration could effectively treat aquaculture wastewater; however, the membrane fouling induced by extracellular organic matter (EOM) during the dewatering process is an issue. This study investigated diatomite dynamic membrane (DDM) fouling behaviour during the dewatering of *Chlorella pyrenoidosa* under the influence of copper ions. The results indicate that copper ion heavy metals in aquaculture wastewater significantly affected purification and algae dewatering by DDM. Aquaculture wastewater with a high copper concentration (1 and 0.5 mg/L) could induce serious DDM fluxes and cake layer filtration resistance (R_c_), whereas fewer filtration fluxes were induced when aquaculture wastewater had a low copper concentration, particularly that of 0.1 mg/L, at which the R_c_ was lowest and the concentration effect was highest. Macromolecular organics of EOM, such as biopolymers, polysaccharides, and proteins, were responsible for DDM fouling and accumulated mostly in the slime layer, whereas only a small amount of them accumulated in the diatomite layer. The DDM rejected more protein-like organics of EOM in the slime layer when dewatering algae at low copper concentrations (<0.1 mg/L); however, when using the DDM to dewater algae at high copper concentrations, more polysaccharides of EOM were rejected (0.5 < Cu^2+^ < 5 mg/L). This result has significant ramifications for aquaculture wastewater treatment as well as algae separation and concentration by the DDM.

## 1. Introduction

Aquaculture water quality has seriously deteriorated due to bait residues and excrement accumulation during the breeding process with the rapid global expansion of aquaculture in recent years. Because excess feeding causes pollution in water bodies, which leads to a series of ecological problems in water areas, careful attention to wastewater treatment is required [1]. Traditional treatment for aquaculture water mainly includes mechanical filtration, foam separation, ozone oxidation, and chlorine dioxide oxidation [2]. Although these processes could effectively remove suspensions, there were some problems, such as high treatment costs, a low reduction in nutrients, and sludge generation [3]; thus, the development of an efficient, low-cost, and environmentally friendly aquaculture wastewater treatment has become very important.

Microalgae are phototrophic, heterotrophic or mixotrophic unicellular microorganisms that are able to convert inorganic nutrients, water, carbon dioxide, and other components into organic substances [4]. Using microalgae to treat sewage could overcome the disadvantages of traditional treatment such as potential losses of nutrients, incomplete utilization of resources, and secondary pollution [5]; the microalgae produced can also be utilized as aquaculture bait [6]. Many studies have used microalgae to purify and treat wastewater, and found that the removal efficiency of nitrogen and phosphate could reach 80% and 85%, respectively, by *Chlorella* [7]. The vital and unavoidable process for microalgae utilization is algae harvesting [8]. Low biomass concentrations and the excretion of algogenic organic matter (AOM) make this harvesting process more difficult, whereas without algae harvesting [9], algae is discharged into the environment, such as with sludge, leading to secondary pollution and significant losses in the economic benefits of water treatment.

Membrane filtration is a promising treatment to harvest algal cells depending on membrane pore size. Zhang et al. studied cross-flow ultrafiltration (UF) to harvest algae cells and found that after filtration, the concentration of *Chlorella* could reach as high as 154.85 g/L, increasing 150 times [10]. Chów et al. found that both microfiltration (MF) and UF could remove cyanobacterial cells [11]. Diatomite dynamic membranes (DDMs) are secondary, formed-in-place membranes that have the advantages of large filtration fluxes and strong anti-fouling abilities [12]. Zhao et al. studied the features of dynamic membrane filtration and indicated that in contrast with traditional membranes, most of the retention of dynamic membranes was conducted by a dynamic cake layer constituted by tiny particles on the potential initial filter surfaces [13]. Because either an UF/MF membrane or the activated sludge in wastewater treatment can be utilized as the underlying primary filter, dynamic membranes have obvious advantages, such as cheap membrane materials, high final algae concentrations, and less frequent backwashing compared with other algal concentration treatments, such as centrifugation, flocculation, sedimentation, and flotation [14].

Nevertheless, the main defects of this technology are membrane fouling, especially the extracellular organic matter (EOM) released by algae during the algal life cycle, which leads to high increases in filtration resistance. Chu et al. investigated the dewatering of *Chlorella pyrenoidosa* using a DDM and found that DDM fouling can be influenced by algal growth phases as well as EOM concentration and compositions [15]. Considering that aquaculture wastewater is different from conventional natural water, a high concentration of heavy metals might exist, as heavy metal and antibiotic additives are commonly utilized during the large-scale breeding process for the treatment and prevention of diseases, promotion of animal growth, and improvement of feed utilization. Whether these heavy metals could affect the purification effects of aquaculture wastewater by microalgae as well as the operation of DDMs during the algal dewatering process remains to be studied.

The purpose of this investigation was thus to study the influence of heavy metals on dynamic membrane fouling and behaviour during the aquaculture wastewater purification and algae dewatering process. Copper ions were selected in this research because copper ions are commonly utilized in additive feed and are frequently detected in aquaculture wastewater and reported at concentrations as high as 9.81 mg/L in livestock and poultry farms in Jiangsu Province, China [16]. This research provides important insights into the purification of aquaculture wastewater by microalgae, as well as microalgae and aquaculture wastewater utilization by dynamic membranes.

## 2. Materials and Methods

### 2.1. Algae Cultivation and EOM Extraction

*C. pyrenoidosa* was purchased from the Institute of Hydrobiology, Chinese Academy of Sciences, and was cultivated in a light incubator at 25 °C and an irradiance of approximately 5000 lx with 14 h of illumination every day. BG11 medium was adopted considering that real aquaculture wastewater contains not only heavy metals but also antibiotics and other substances, which influence algal growth. The copper concentration was set at 0.01, 0.05, 0.1, 0.5, 1, and 5 mg/L.

EOM was obtained by centrifugation of the algal cells at 6000 rpm for 15 min and then filtrating through a 0.45 μm filter to remove the algal cells [17].

### 2.2. Experimental Procedure

**Figure 1:**
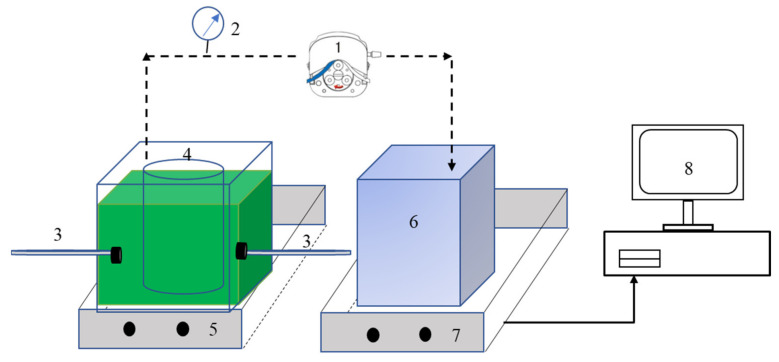
Schematic diagram of the filtration setup: (**1**) peristaltic pump; (**2**) pressure gauge; (**3**) air supply; (**4**) membrane module; (**5**) magnetic stirrer; (**6**) effluent tank; (**7**) electronic balance; (**8**) computer.

The experimental system used for algae concentration is shown in Figure 1. The DDM system was composed of a cylindrical membrane module, an aeration system, and a feed pump. The dynamic membrane module used was a cylindrical sintered polyethylene (PE) filter tube, which had a relatively high filtration pressure tolerance and good corrosion resistance to acid and alkali solvents. The thickness of the module was 3 mm, with an average aperture of 200–300 mesh and a total filtration area of 109 cm^2^. The diatomite utilized had an average particle size of 7.8 μm, with its BET specific surface area of 59.4246 m^2^/g (Zhejiang, China).

Prior to operation, precoating was performed using a peristaltic pump at full speed for 15 min until a cake layer was deposited on the support membrane, and the turbidity of the effluent was <1.0 NTU. Then, the concentration of algae was determined at a flow rate of 26 mL/min at 25 °C and pH 7. During concentration, all the filtration flux and transmembrane pressure (TPM) values were recorded, and the filtration time was 60 min. Prior to filtration, all algae concentrations were adjusted to an OD_680_ of 0.6, and the diatomite dose was 25 g/L. The aeration rate was 4 m^3^/m^2^ h. When filtration was completed, air-water backwash was performed to remove the cake layer, restoring the membrane flux. All experiments were repeated three times, and the mean and standard error of triplicate experiments were calculated by software SPSS 9.0. The correlation between membrane fouling and water quality was evaluated by principal component analysis (PCA) using SPSS 9.0.

### 2.3. Analytical Methods

The algal *C. pyrenoidosa* cell concentration was determined using OD_680_. EOM was characterized by the determination of molecular weight (MW) distribution, fluorescence excitation-emission matrix (EEM) spectra, total organic carbon (TOC), and ultraviolet absorbance at a wavelength of 254 nm (UV_254_). Total nitrogen (TN) was determined by the potassium persulfate-ultraviolet absorption method. TOC and UV_254_ were measured by a TOC analyser (TOC-VCPH, Shimadzu, Japan) and a dual-beam spectrophotometer (DR-5000, Hach), respectively.

The MW distribution was measured using high-performance size exclusion chromatograph (HPSEC) with ultraviolet absorbance (UVA) and online TOC analysis. The overlapping peaks and the peak areas were evaluated by the peak-fitting technique [18].

EEM spectra were acquired via a fluorescence spectrophotometer (Hitachi, F-2000), the excitation (Ex) and emission (Em) wavelength were set at 220–550 nm, and parallel factor analysis (PARAFAC) of EEM was conducted using DOMFluor Toolbox software in MATLAB^®^ to obtain the maximum fluorescence intensity (FI) of each component [19].

When filtration was completed, the cake layer of DDM was divided into three sublayers using a medical scalpel and cut into small pieces of 1 cm × 1 cm according to previous research: the slime layer, the algae layer, and the diatomite layer [15]. The contents of protein and polysaccharides in the three sublayers were measured by means of the modified Lowry method and the enthrone-sulfuric acid method, respectively [20].

The filtration resistance of the cake layer (R_c_) can be obtained using Equation (1)

(1) $$R_{c}=\frac{\Delta P}{\mu j}-R_{m}$$

where ΔP (Pa), μ (Pa s), and j (m/s) represent the TMP, the viscosity of the permeate, and the filtration flux, respectively, and R_m_ is the filtration resistance of the support membrane.

Meanwhile, the rejection rate of organic matter (β) can be obtained:

(2) $$\beta =\frac{C_{F}-C_{P}}{C_{F}}$$

where $C_{F}$ and $C_{P}$ represent the organic concentration of feed and permeate water.

## 3. Results and Discussion

### 3.1. Influence of Copper Ions in Aquaculture Wastewater on DDM Filtration Performance

**Figure 2:**
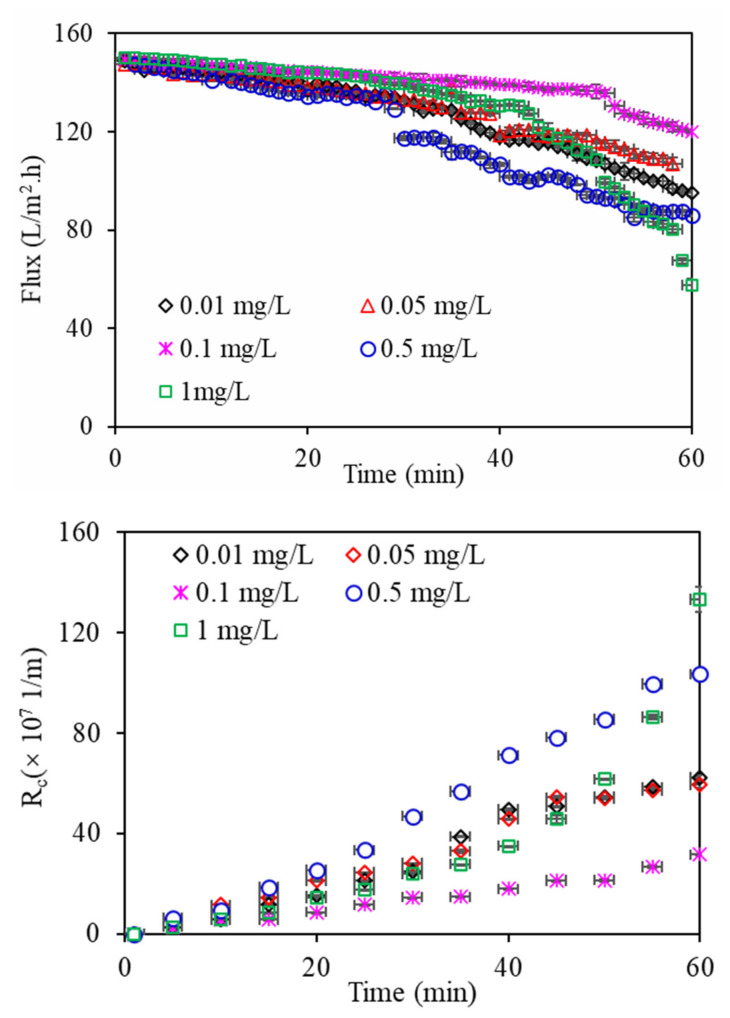
DDM flux and R_c_ variations of algae under various copper concentrations.

Figure 2 shows the filtration flux changes in the DDM by algae under various copper concentrations. All the filtration flux values of algae declined slowly in the primary filtration process and then rapidly, which can be explained by increased blockage and adjustment of the DDM pore size; however, with increasing filtration time, the algae particles that were bigger than the membrane pore size gradually accumulated on the membrane surface and constituted the algae layer, leading to a significant decrease in filtration flux. In addition, the filtration flux of the DDM changed significantly for all the algae under various copper concentrations. Algae exposed to 0.1 mg/L Cu^2+^ had the smallest decline in filtration flux, whereas the filtration flux declined severely with increasing copper concentration, especially at Cu^2+^ 1 mg/L, which can be interpretated as the presence of copper ions in aquaculture wastewater influencing microalgal growth during the algal purification of aquaculture wastewater (Figure S1). It had been accepted that the first flux decrease is related to pore blockage, while the second flux decline is mainly related to cake formation. Algae exposed to 1 mg/L Cu^2+^ had the most serious decline, which can be explained that algae exposed to 0.1 mg/L Cu^2+^ might form a tighter cake layer on the membrane surface, resulting in higher resistance due to its higher macro molecular organics in EOM. The characteristics of released EOM can also be changed simultaneously, thereby altering their DDM fouling behaviours. Huang et al. investigated the ultrafiltration (UF) fouling behaviour of algal EOM and intracellular organic matter (IOM) of *Microcystis aeruginosa* when using UF to treat algae-laden water, and found that both EOM and IOM induced the most serious reductions in filtration flux at the lowest copper concentration of 0.01 μmol/L, followed by EOM at high copper concentrations of 0.3 and 0.1 μmol/L [21]. This result was slightly different from our observation, which might be explained by the concentration of copper ions as well as the types of algae, membrane and water utilized. Notably, since the algae died in the aquaculture wastewater containing a high copper concentration of 5 mg/L, the filtration flux of algae exposed to 5 mg/L copper was not investigated.

The R_c_ changes in algae solutions under various copper concentrations were also investigated (Figure 2). Similar to the flux changes, algae exposed to 0.1 mg/L Cu^2+^ had the lowest R_c_, followed by 0.05, 0.01, 0.5, and 1 mg/L Cu^2+^, indicating that the DDM fouling behaviour can also be affected by the copper concentration in aquaculture wastewater. A relatively high copper ion concentration in aquaculture wastewater causes serious filtration fluxes and a high R_c_ value, whereas a lower R_c_ value and decreases in fouling and filtration flux would be induced when algae are present at low copper concentrations. Previous research suggested that R_c_ can be separated into thickness-increase resistance and compaction resistance [22]. The support membrane used in this study had an average aperture of 200–300 mesh, equal to a pore size of 75 mm; therefore, the filtration resistance of the support mesh could be ignored, while the thickness of DDM was much thicker than the cake layer formed by algae on the membrane. The discrepancies of R_c_ thus originate either from the thickness-increase resistance in the primary filtration process and/or the compaction resistance when the shear force was greater than the drag force for large particle surfaces. Notably, since there were great discrepancies between the experimental R_c_ values on the membrane surface and the hydraulic resistance calculated by the Kozeny–Carman equation according to previous research [23], the filtration process using new osmotic pressure theory needs to be further investigated.

Moreover, it was found that during the whole dewatering process, no membrane fractures occurred for any of the algae under various copper concentrations (Figure S2), which was in contrast with the results of Chu et al. [22], suggesting that the cylindrical sintered PE filter tube used in this study is much more superior in stability as a support than the stainless steel support mesh, as previous research indicated.

### 3.2. Influence of Copper Ions in Aquaculture Wastewater on Algal Concentration Effects

**Figure 3:**
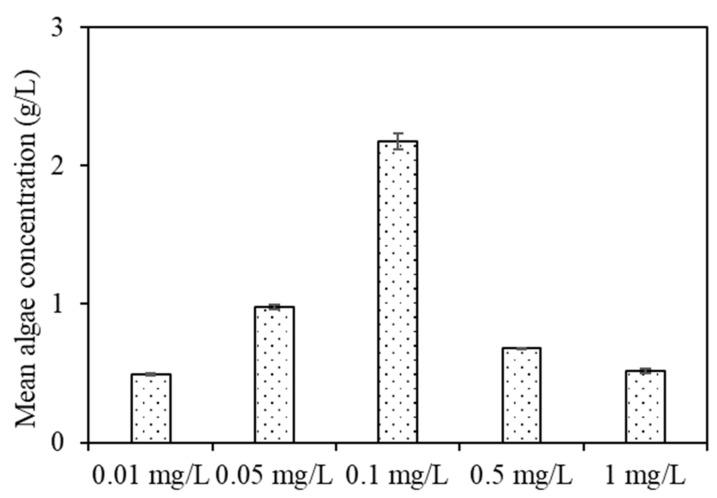
Actual dewatering effects of algae under various copper concentrations by DDM.

Figure 3 shows the algae concentrations of feed water after DDM filtration. There were large discrepancies in algae concentration among the samples with various copper ion concentrations subjected to DDM filtration. The actual algae concentration was 2.174 g/L for algae subjected to 0.1 mg/L copper, compared with 0.489, 0.978, 0.6782, and 0.517 g/L algae observed in the 0.01, 0.05, 0.5, and 1 mg/L copper solutions, respectively, illustrating that algae subjected to 0.1 mg/L copper was more abundant than those exposed to other copper concentrations when the copper concentration was <0.1 mg/L. Moreover, the concentration effects increased with an increasing copper concentration; however, when the copper concentration was >0.1 mg/L, the concentration effects gradually decreased. This phenomenon can be ascribed to the EOM concentration and characteristics that might have affected DDM filtration behaviour, as well as algal separation and concentration effects. Zhang et al. investigated the dynamic membrane filtration characteristics and suggested that dynamic membrane filtration has obvious advantages in terms of cost, energy consumption and concentrated solid concentration compared with other concentration methods; yet, the main issues are membrane fouling, especially serious fouling induced by EOM deposited on the membrane surface, rather than algal cells [24]. Xing et al. found that natural organic matter characteristics had a great influence on ultrafiltration and microfiltration membrane fouling [25]. To further investigate the DDM filtration and dewatering behaviours of algae exposed to various copper concentrations, the organic removal and EOM characteristics before and after DDM filtration were further identified.

### 3.3. Organic Removal of EOM under Various Copper Concentrations by DDM

**Figure 4:**
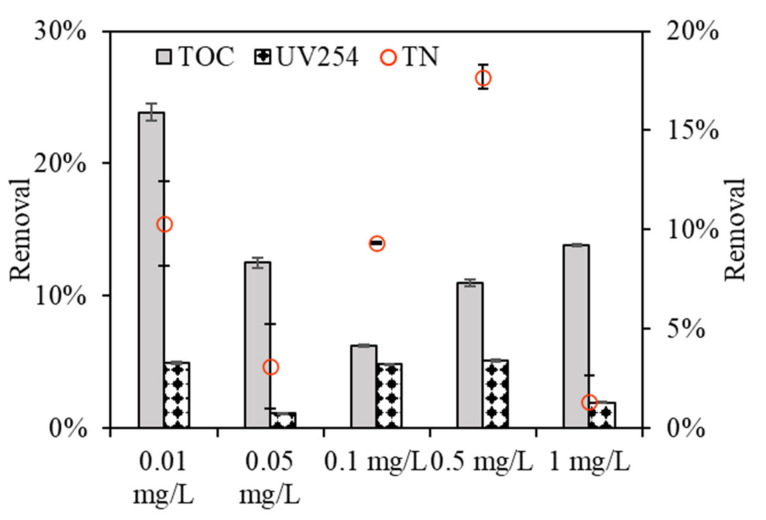
Organic removal of algae under various copper concentrations by DDM.

Figure 4 depicts the organic rejection of algae by the DDM with various copper concentrations. Algae exposed to 0.1 mg/L copper had relatively low TOC removals, with removal efficiencies of 6.21%, compared to removal efficiencies of 23.88%, 12.5%, and 10.96%, and 13.84% for algae exposed to 0.01, 0.05, and 0.5 and 1 mg/L copper, respectively, which had some discrepancies with their DDM filtration fluxes (Figure 2). This phenomenon can explained be the fact that the support membrane used in this study had an average aperture of 200–300 mesh, equal to a pore size of 75 mm, despite the diatomite layer and algae layer which form during DDM dewatering, as only part of the macro-organic matter can be rejected, whereas most of the organics permeated (please see Table S1 in the supporting information), and the rejection rate was low. Notably, all the UV_254_ rejections were lower for EOM than TOC removals under various copper concentrations, and the relatively low UV_254_ reduction by DDM filtration suggests that UV-absorbing organics are much more difficult to reject using DDMs.

From the TN rejections during DDM filtration, it was found that DDM exhibited high nitrogen removal when algae was exposed to a low copper concentration of 0.01 mg/L; however, the removal efficiency decreased with increasing copper concentration, and the removal efficiency was 9.5%, 5.2%, 1.2%, 0.6%, and 2.7% for algae exposed to 0.01, 0.05, 0.1, 0.5, and 1 mg/L copper, respectively, implying that DDM filtration also had extremely different TN rejection rates when algae was exposed to various copper concentrations. Zhang et al. investigated the structure and effect of dynamic membranes in a dynamic membrane bioreactor and found that the average TN removal rate was 6% [26]. The diversity of TN rejections by DDM filtration lies in the rejections of algae and associated organic matter, thus leading to the variation in inorganic matter retention.

### 3.4. Analysis of EOM Characteristics during Aquaculture Wastewater Purification

**Figure 5:**
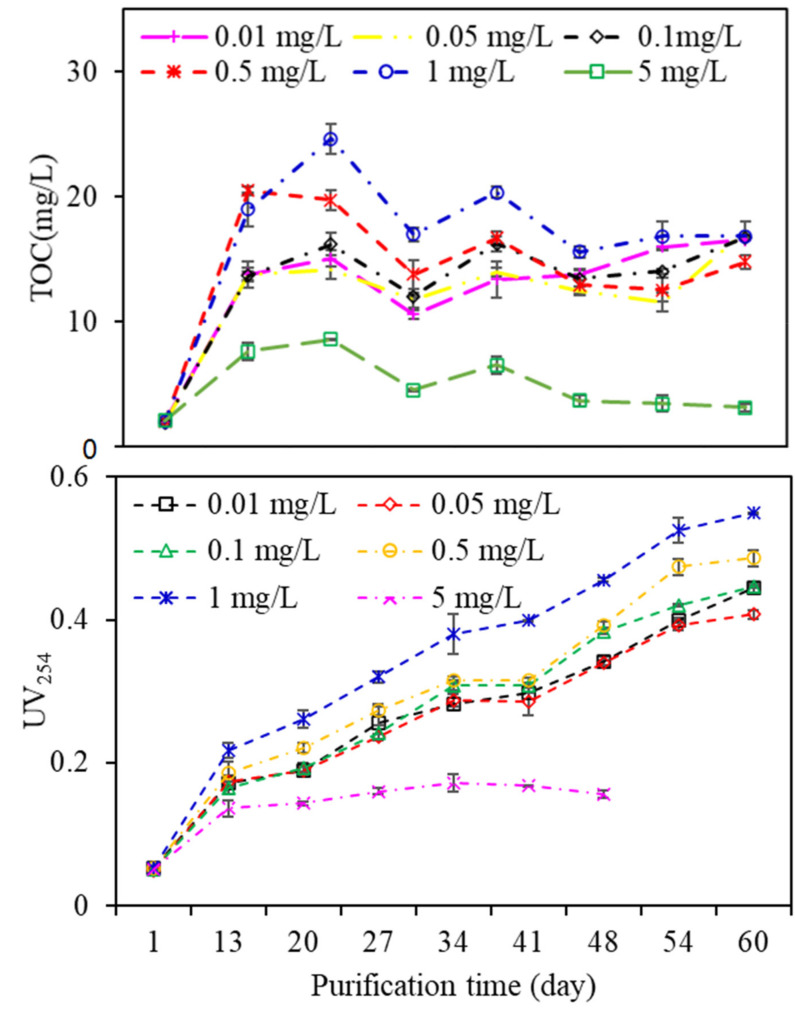
Fluctuations in TOC and UV_254_ during the wastewater purification process.

Bound and dissolved EOM released by algae are considered the main causes of membrane fouling, and filterability declines as the EOM content increases [27]. Figure 5 shows the fluctuations in TOC and UV_254_ during the wastewater purification process. All the TOC and UV_254_ values increased gradually, except for that of algae exposed to 5 mg/L copper, while there were great fluctuations in TOC and UV_254_ values among EOM from algae exposed to other copper concentrations. EOM with 1 mg/L copper exposure had the highest TOC content during the purification process, followed by 0.5, 0.1, 0.05, and 0.01 mg/L copper, which was extremely different from that of algal growth in Figure S1; that is, algae exposed to a low copper concentration (0.01 mg/L) had the highest growth rate, whereas the algal growth gradually decreased with increasing copper concentrations, especially when the copper concentration reached 5 mg/L, at which the algae died after growing for a period of time. This phenomenon can be explained by the fact that microscale copper ions are vital for algae metabolism, but are toxic at high concentrations. Since copper ions are a strong inhibitor of cell metabolism, during long-term resistance, algae have established a series of adaptive mechanisms, such as combining with heavy metals through extracellular components, synthesizing metal-binding proteins or polypeptides, and combining with relevant ligands in algal cells [28]. EOM exposed to 1 mg/L copper had the highest UV_254_ value, whereas the lowest UV_254_ value occurred in the EOM subjected to 0.01 mg/L copper, suggesting that although a high copper concentration in aquaculture wastewater did not facilitate the growth of algae, the total organic matter as well as UV-absorbing substances are preferably released during the algal purification process, which subsequently leads to the observed influence on DDM filtration.

**Figure 6:**
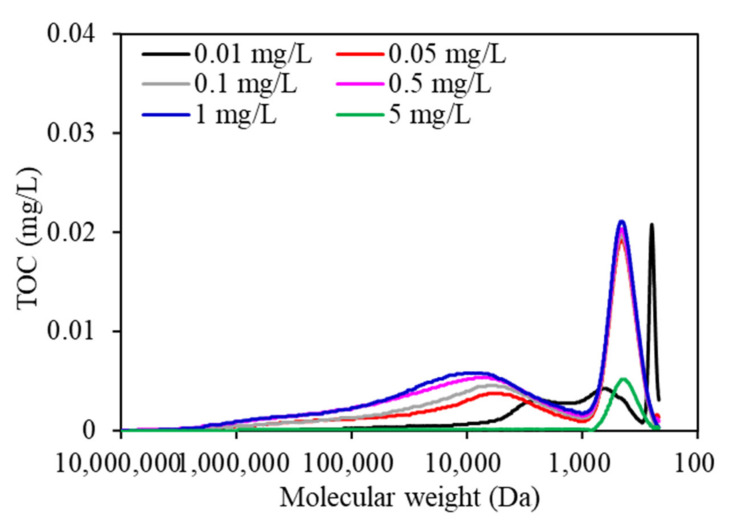
Molecular weight distribution of EOM under various copper concentrations during purification process.

As shown in Figure 6, the MW distribution of EOM during the whole wastewater purification process changed significantly. Three peaks were found for all EOM samples according to TOC chromatography. Peak A, which had a high TOC peak area, was reported to be associated with biopolymers (i.e., polysaccharides, proteins, or amino sugars), and peak B and peak C were associated with humic-like substances (HSs) and building blocks of low-MW acids and humic acids, respectively [29]. EOM exposed to 1 mg/L copper had the highest TOC peak areas, followed by that exposed to 0.5, 0.1, 0.05, 0.01, and 5 mg/L copper, whereas the TOC peak areas in low-MW regions demonstrated similar trends, with the TOC peaks in EOM subjected to 1 mg/L copper being the highest, followed by 0.5 > 0.1 > 0.05 > 0.01 mg/L copper, suggesting that the organic composition of EOM can also be changed when aquaculture wastewater contains various copper concentrations during the purification process. More macromolecular and small-molecule organics, such as proteins, polysaccharides or amino sugars, and HSs were more likely to be synthesized in EOM when aquaculture wastewater contained copper at a high concentration, which leads to serious membrane fouling following DDM filtration (Figure 2), whereas fewer macromolecular and small-molecule organics were synthesized when algae were exposed to low copper concentrations of 0.01 and 0.05 mg/L. Xi et al. studied the effects of copper ions on the protein, polysaccharide, and malondialdehyde (MDA) contents of *C. vulgaris*, and found that copper ions at low concentrations had obvious effects on intracellular polysaccharide secretion; however, the membrane structure can be severely damaged at high copper concentrations, which leads to an increase in membrane permeability and penetration of the intracellular electrolyte [30].

**Figure 7:**
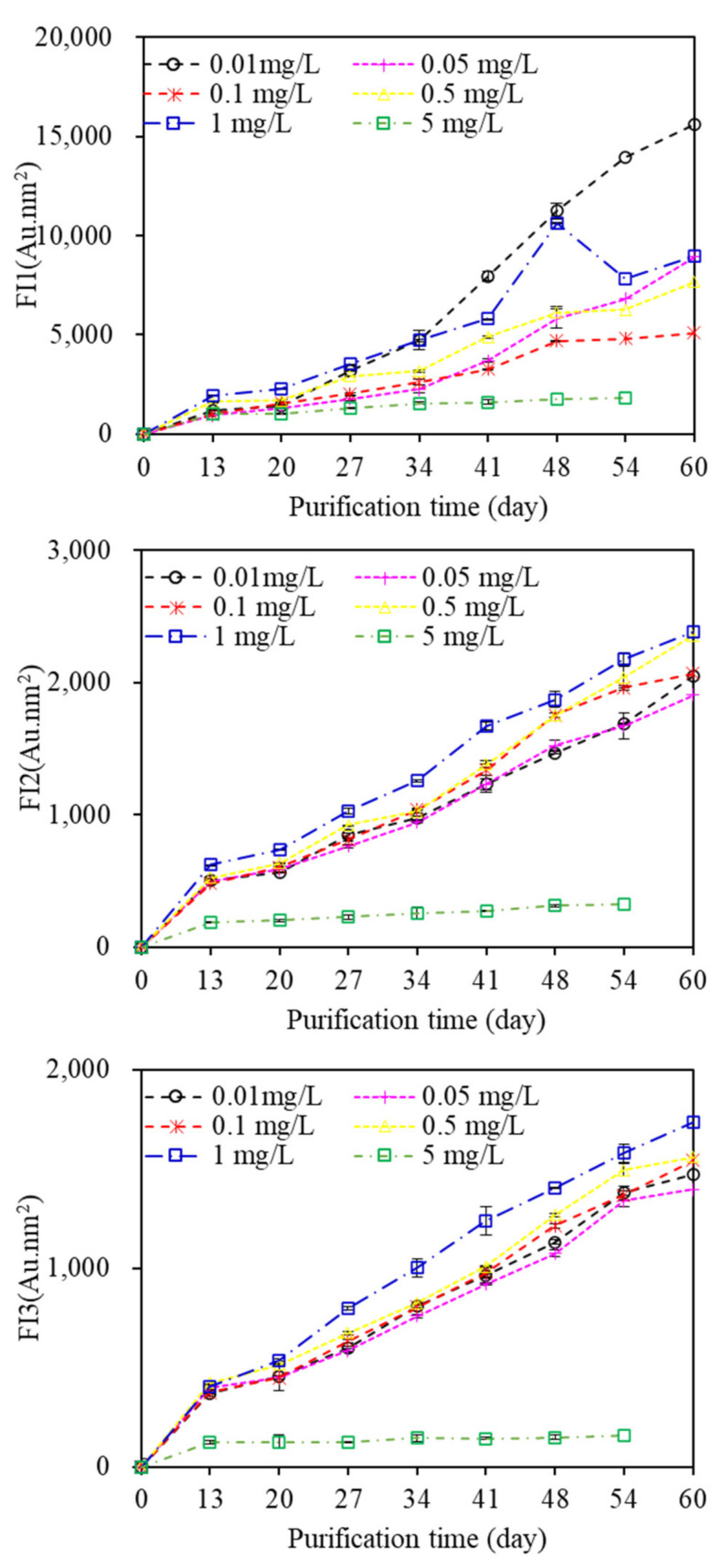
Fluorescence intensities of EOM under various copper concentrations during purification by algae.

Fluorescent EOM was also reported to contain a large quantity of aromatic constructions and unsaturated fatty chains with different kinds of fluorescent functional groups [31]. Previous research suggested that fluorescent EOM can be utilized for the analysis of membrane foulants, which provided specific information on protein and HSs or fulvic-like substances [29]. Figure S3 displays the EEM spectra of EOM during the aquaculture wastewater purification process. Three fluorescent components can be identified through EEM-PARAFAC. Component 1, which had maxima at Em and Ex wavelengths of 330 and 275 nm, respectively, represented protein-like fluorescence (tryptophans); component 2, which exhibited maxima at Ex and Em wavelengths of 250 and 325/385 nm, respectively, indicated HS fluorescence; and component 3 (Ex/Em = 260, 350/450 nm) indicated fulvic acid-like substance fluorescence. The FIs of all components increased gradually with increasing purification time (Figure 7), indicating that fluorescent organic matter, such as protein and humic acid, constantly migrated to wastewater when algae were utilized to purify the aquaculture wastewater, and there were large differences in the FIs of components among EOM samples exposed to various copper concentrations. Algae exposed to 0.1 mg/L copper had the lowest FI of component 1, whereas the highest FI of component 1 occurred with 0.01 mg/L copper exposure, suggesting that algae exposed to the lowest copper concentration of 0.01 mg/L would release more fluorescent protein-like substances, whereas high copper concentrations promote algae to release fluorescent HSs, which was in line with the observed MW distributions. In addition, the FI of component 1 was much higher than those of components 2 and 3, suggesting that protein-like organics were more likely to be released than HSs during the whole aquaculture wastewater purification process.

From the TN during the purification process (Figure S4), TN contents in aquaculture wastewater were found to be 64.56, 60.3, 67.86, 66.24, 68.22, and 96 mg/L when algae were exposed to 0.01, 0.05, 0.1, 0.5, and 1 mg/L copper, respectively, at the end of purification, indicating that algae exposed to low copper concentrations were more able to remove TN, which was in accordance with their algal growth (Figure S1).

### 3.5. Analysis of Fouling Behaviour during the Algae Dewatering Process by DDM Filtration

**Figure 8:**
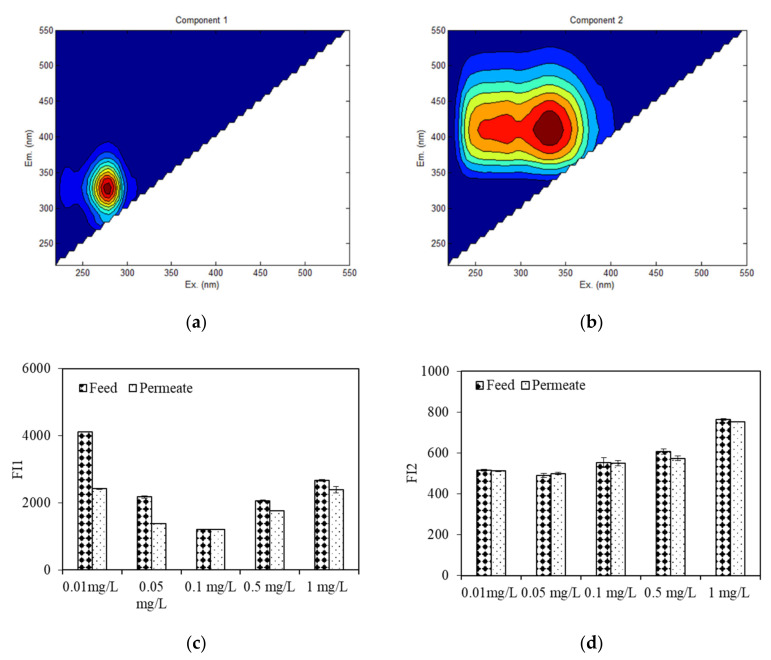
EEM spectra and variation of fluorescence intensity of feed and permeate by DDM using EEM-PARAFAC, (**a**,**b**) EEM contour of components; (**c**,**d**) fluorescence intensity of feed and permeate by EEM.

Protein-like organics were reported to be major components that would cause serious membrane fouling [17]. Figure 8 depicts the EEM spectra of feed and permeate water during algae dewatering by DDM filtration using EEM-PARAFAC. Unlike the EEM spectra of EOM during the wastewater purification process, there were only two fluorescent components of the feed and effluent water during the dewatering process (Figure 8a,b), due to the smaller numbers of water samples as well as lower organic concentrations. EOM exposed to 0.01 mg/L copper had the highest FI of protein-like component 1 (Ex/Em = 275 nm/330 nm), followed by those of EOM exposed to 0.05, 0.5, 1, and 0.1 mg/L copper, while the highest FI of HS fluorescence (Ex, Em = 275, 345 nm/400 nm) occurred with 1 mg/L copper exposure, indicating that when the algae concentration was held constant, the EOM of algae exposed to low copper concentrations contain more protein-like organics than that of algae exposed to higher copper concentrations, whereas more HSs in EOM were observed when algae were exposed to high copper concentrations.

After dewatering by DDM filtration (Figure 8c,d), the FI protein-like component was found to be greatly decreased, especially when algae were treated with copper at 0.01 and 0.05 mg/L, the removal efficiencies were as high as 41% and 36%, respectively, whereas there were small fluctuations in the HS FIs among the EOM samples from algae exposed to various copper concentrations, which was in line with the UV removal results (Figure 4). This result suggests that fluorescent protein-like organics are the major components that were intercepted by the DDM, especially when the aquaculture wastewater contained a low copper concentration (<0.1 mg/L); however, fluorescent HSs and fulvic acid-like substances were rarely retained. Chu et al. investigated the EEM spectra of a DDM and found that after a long period of operation, the slime and algae layers mainly retained protein-like organics, while HSs are mainly retained by the algae and diatomite layers [15]. The difference in our result lie in the algae dewatering time. EOM with low copper exposure (0.01 mg/L and 0.05 mg/L) had relatively high rejection of fluorescent protein-like organics, which was different from the observed DDM flux behaviours (Figure 1). This result can be interpretated by the reality that other organics, such as polysaccharide-like substances, can also be major components that lead to serious DDM fouling; however, most polysaccharide-like organics were reported to have no fluorescent characteristics [19].

Table S1 shows the MW distribution of EOM before and after DDM filtration. Similar to the MW distribution of EOM during the algae purification process, there were large differences in MW distribution with varying copper concentrations in the feed water. EOM with 1 mg/L copper exposure had the highest TOC content and highest peak A area, followed by those of EOM with 0.5, 0.01, 0.05, and 0.1 mg/L copper exposure, with TOC contents of 2.18, 2.12, 1.50, 3.18, and 5.14 mg/L, respectively, which was in line with their filtration fluxes. In addition, the TOC peak areas of medium-MW organics followed the order of 0.5 > 1 > 0.01 > 0.05 mg/L > 0.1 mg/L copper, suggesting that when the DDM filtered the algae at the same concentration, EOM at high copper concentrations (0.5 and 1 mg/L) also had the highest medium-MW organic compound content.

For microalgae dewatering, the TOC peak areas of macromolecular biopolymers were greatly reduced after dewatering, with rejection rates of 76.99%, 13.89%, 1.3%, 15.96%, and 31.47% for EOM exposed to copper concentrations of 0.01, 0.05, 0.1, 0.5, and 1 mg/L, respectively. Moreover, medium-MW HSs were also partly removed, and the removal efficiencies were 62.83%, 0%, 11.85%, 25.43%, and 3.29% for EOM exposed to copper concentrations of 0.01, 0.05, 0.1, 0.5, and 1 mg/L, respectively, indicating that macromolecular biopolymers and part of the medium-MW HSs were the major components that caused DDM fouling during the algae dewatering process. Previous research suggested that size exclusion was a key mechanism for membrane filtration. Organics nearing the membrane pore size lead to serious pore blockage, making the filtration resistance enhanced, whereas cake formation is induced by the organics with sizes greater than membrane pores [29]. The high-MW substances that were rejected during the dewatering process can be explained by the slime layer and the algae layer of the DDM having high rejection rates of these organic substances.

**Figure 9:**
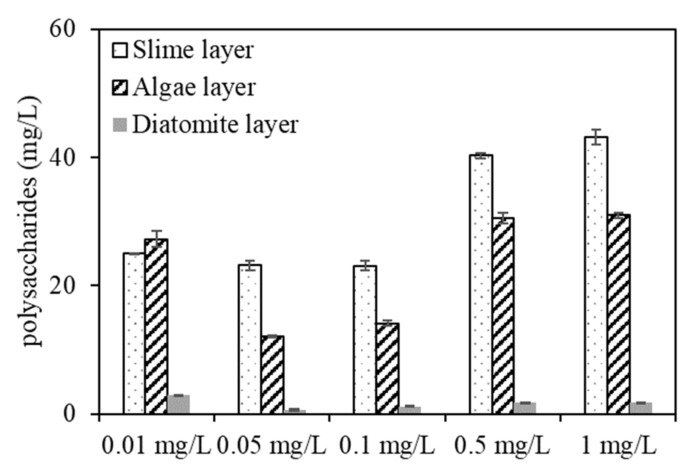
Protein and polysaccharide content in the three sublayers.

To further study the DDM filtration and fouling behaviour of algae under various copper concentrations, the protein and polysaccharide contents in the three DDM sublayers were further identified (Figure 9). Figure 9 shows that most protein and polysaccharide compounds accumulated in the slime layer, whereas a small amount of EOM cumulated in the diatomite layer. The protein contents in the slime layer were 31, 27.8, 15.05, 19.89, and 21 mg/L for algae exposed to copper concentrations of 0.01, 0.05, 0.1, 0.5, and 1 mg/L, respectively, indicating that the DDM would retain more protein organics in the slime layer when dewatering the algae under low copper concentrations, whereas a lower protein content was rejected by the DDM when filtering the algae with a copper concentration of 0.1 mg/L, which was in accordance with the EEM result (Figure 8).

The polysaccharide contents in the three sublayers were much higher with high copper concentrations (0.5 and 1 mg/L) than with low copper concentrations, whereas the contents were comparatively low under low copper concentrations, and the polysaccharides contents in the slime layer were 25.12, 23.19, 23.14, 40.26, and 43.18 mg/L for algae exposed to copper at 0.01, 0.05, 0.1, 0.5, and 1 mg/L, respectively, and were 27.3, 12.03, 14.15, 30.56, and 31 mg/L for polysaccharides in the algae layer, indicating that polysaccharides within the slime and algae layers are also key factors causing DDM R_c_ fouling, which can be considered as one of the explanations for the filtration flux decreases seen in Figure 1. Notably, since polysaccharides in EOM have been proven to be mainly hydrophilic, while the diatomaceous utilized in the experiment was hydrophilic, the rejection of polysaccharide organics by the diatomite layer was slightly higher than that of protein contents, which was consistent with previous research [29].

### 3.6. Discussion of Organic Components and DDM Fouling with Various Copper Concentrations

**Figure 10:**
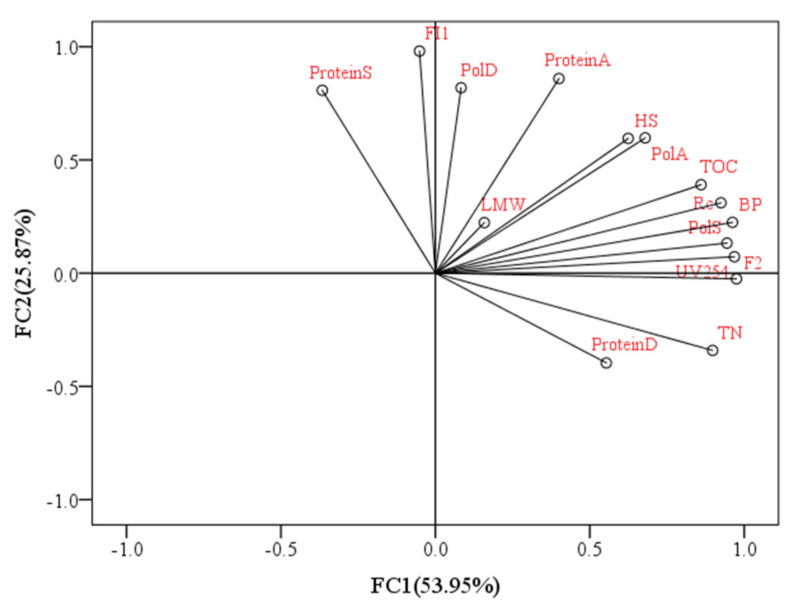
PCA of R_c_ and feed water qualities.

Aquaculture wastewater treatment is of great concern due to its negative ecological and environmental impacts. Combined microalgal and membrane filtration could effectively treat aquaculture wastewater; however, the main problems are algae dewatering, especially the membrane fouling induced by EOM during the algal dewatering process. Many studies have investigated the fouling behaviours correlating with water characteristics to diagnose membrane fouling [32,33]. Although the DDM has obvious advantages compared with other algal concentration treatments, the main problem is membrane fouling; therefore, effectively analysing the relationship between organic characteristics and DDM fouling is very important for DDM operation and purification during the algal dewatering process. Figure 10 depicts the relationship between DDM membrane fouling (R_c_) and the raw water characteristics of EOM under various copper concentrations (DOC, UV_254_, biopolymers, HSs, low-MW (LMW) compounds, FI1, FI2, polysaccharides and protein). Pearson’s correlation matrix was utilized for this analysis. It can be seen from Figure 10 that 79.8% of the data variance was explained by the first two principal components, which indicated good explanations of the data. In addition, there were high correlations between R_c_ and macromolecular organic biopolymers (r^2^ = 0.968), which indicated that biopolymers contributed greatly to DDM fouling during the algal dewatering process, which was consistent with previous research suggesting that biopolymers (biopolymers), polysaccharides, and proteins were mainly responsible for membrane fouling [34]. Previous research suggested that hydrophilic (HPI) organics, which usually contain lots of polar functional groups, had stronger affinity towards water than HPO organics [35], as HPI organics could form hydrogen bonds with water molecules, and they had lower tendency to adhere to the membrane; however, when these HPI organics formed a cake layer on the membrane surface, they caused greater resistance to water flow than hydrophobic (HPO) organics due to stronger foulant–water interaction, whereas HPO organics had greater tendency to adhere to the membrane and aggravate the irreversible adhesion. Similar to the observed biopolymers, the TOC also clustered with R_c_, exhibiting high correlation (r^2^ = 0.888, *p* = 0.044) and revealing that the TOC content in algal solutions was also extremely relevant with fouling potential during the algal dewatering process. The argument that the low molecular components contributed less to membrane fouling can be sustained by the minimal correlation of R_c_ with low-MW (LMW) acids and humic acids, with an r^2^ of 0.049 (*p* = 0.937).

For HSs, a certain amount of HS organics was found in the extracted substances, with the correlation of R_c_ with HSs being r^2^ = 0.871. HSs were reported to be some of the main foulants in low-pressure membrane filtration performance according to previous research [32]. As HSs were also partly removed by the DDM in this study (Table S1), HSs may also play some roles in the formation of membrane fouling during DDM operation. Similarly to the observed TOC, the UV_254_ values also had comparatively high correlations with R_c_ (r^2^ = 0.865). UV_254_ could be utilized as a good surrogate parameter for HSs in most cases, as some of the HSs can be rejected by the DDM and some can be retained (Figure 4), which further indicated that HSs is important in the formation of membrane fouling (Table S1). For fluorescent components 1 and 2, the r^2^ values were 0.226 and 0.845 for FI1 and FI2, respectively, indicating that fluorescent protein-like organics had little correlation with DDM fouling during the dewatering process. It should be noted that although polysaccharides were reported to be mainly responsible for membrane fouling [36], there were great discrepancies in polysaccharide contents in the DDM correlated with R_c_. The polysaccharide components in the slime layer (PolS) contributing greatly to membrane fouling can be demonstrated by the strong correlation with R_c_ (r^2^ = 0.950, *p* = 0.13), whereas less fouling is contributed by polysaccharides in the algae layer (PolA) and diatomite layer (PolD) as indicated by the smaller correlations with R_c_, with r^2^ values of 0.799 and 0.237, respectively, which indicated that polysaccharides in the slime layer may be more suitable as indicators of membrane fouling in the DDM. This phenomenon can be explained by the fact that the slime layer has the densest construction, which is responsible for filtration resistance [18], followed by the algae layer and diatomite layer, with the polysaccharide concentration reducing gradually. The protein content in the DDM had a lower correlation with R_c_ than the polysaccharide content, and the r^2^ values were −0.033, 0.700, and 0.479 for proteins in the slime layer (ProteinS), algae layer (ProteinA), and diatomite layer (ProteinD), respectively.

Based on this analysis, the influence of copper ions in aquaculture wastewater on DDM fouling during the dewatering of *C. pyrenoidosa* can be illustrated as follows: macromolecular biopolymers and polysaccharides in the slime layer were highly associated with DDM filtration. Combined algae and DDM filtration have excellent effects on organic and inorganic rejections during aquaculture wastewater purification; however, as copper ions existed in aquaculture wastewater, the purification effects and algae dewatering were significantly affected. A high concentration of copper ions in aquaculture wastewater significantly reduces the growth of microalgae during the purification process, yet the total organic matter and macromolecular organic matter synthesized in EOM were the highest, leading to subsequent decreases in DDM filtration fluxes as well as algae dewatering effects. When aquaculture wastewater contained low copper concentrations (≤0.1 mg/L), despite the algal growth being greatly promoted, the total organic matter and macromolecular organics synthesized were reduced, thus leading to the alleviation of DDM fouling, especially when algae were exposed to 0.1 mg/L copper. At this copper concentration, the R_c_ value was lowest, while the algae concentration effect was highest. Therefore, effective removal of copper ions in aquaculture wastewater before aquaculture wastewater utilization not only promotes the growth of economic microalgae, but also ameliorate DDM filtration behaviours and algae dewatering effects.

## 4. Conclusions

In this study, the effects of copper ions in aquaculture wastewater on DDM fouling and behaviour during dewatering of *C. pyrenoidosa* were investigated, and the key findings were as follows:

Copper ions in aquaculture wastewater had significant effects on water purification and algae dewatering by DDM filtration. Aquaculture wastewater containing high copper concentrations of 1 mg/L and 0.5 mg/L induces serious DDM fluxes and R_c_, whereas lower filtration fluxes were induced when aquaculture wastewater contained low copper concentrations (≤0.1 mg/L), especially at copper concentration was 0.1 mg/L. At this concentration, the DDM R_c_ was lowest, whereas the concentration effect was highest.

More total organic matter and UV-absorbing substances were released when aquaculture wastewater contained a high copper concentration of 1 mg/L during the algal purification process, whereas aquaculture wastewater containing a low copper concentration of 0.05 mg/L reduced the production of EOM and UV-absorbing organic compounds. Macromolecular biopolymers, polysaccharides and proteins of EOM were mainly responsible for DDM fouling during the algae dewatering process and accumulated mostly in the slime and algae layers, and only a small amount of them accumulated in the diatomite layer. DDM retained more protein-like organics of EOM in the slime layer than in the other two layers when dewatering algae at low copper concentrations of 0.01 and 0.05 mg/L, whereas more polysaccharides in EOM were rejected when DDM filtration with algae was implemented with high copper concentrations of 0.5 mg/L and 1 mg/L. These results have important significance to aquaculture wastewater treatment as well as algae separation and dewatering by DDM filtration.

## Data Availability

Not applicable.

## Supplementary Materials

The following are available online at https://www.mdpi.com/article/10.3390/membranes11120945/s1, Figure S1: Growth of algae under various copper concentrations during aquaculture wastewater treatment, Figure S2: Schematic diagram of the dynamic membrane structure, Figure S3: EEM spectra of EOM under various copper concentrations during the purification process by EEM-PARAFAC, Figure S4: Total nitrogen content of algae under various copper concentrations after purification, Table S1: Peak area of DOC fraction of EOM under various copper concentrations and organic removal by DDM via HPSEC-TOC-UV combined with peak-fitting.
