# Supplementary material for: Diatomite Dynamic Membrane Fouling Behaviour during Dewatering of Chlorella pyrenoidosa in Aquaculture Wastewater
Source: Membranes (Basel). 2021 Nov 29;11(12):945. doi: 10.3390/membranes11120945 (PMC8706875; doi:10.3390/membranes11120945)
Supplement: Supplementary file 1 [file membranes-11-00945-s001.zip › membranes-1467330-supplementary.pdf]

membranes

Supplementary Material

Diatomite Dynamic Membrane Fouling Behaviour during Dewatering of *Chlorella pyrenoidosa* in Aquaculture Wastewater

Weiwei Huang [a,b], Weiguang Lv[a], Huaqiang Chu [b], Weiwei Lv [a,c] and Wenzong Zhou [a,*], Bingzhi Dong [b]

[a] Eco-Environment Protection Research Institute, Shanghai Academy of Agricultural Sciences, Shanghai 201403, China
[b] School of Environmental Science and Engineering, Tongji University, Shanghai 200092, China
[c] Shanghai Runzhuang Agricultural Technology Co., LTD, Shanghai 201403, China

Table S1. Peak area of DOC fraction of EOM under various copper concentrations and organic removal by DDM via HPSEC-TOC-UV combined with peak-fitting.

EOM under Cu^{2+}	0.01 (mg/L)	0.05 (mg/L)	0.1 (mg/L)	0.5 (mg/L)	1 (mg/L)
Peak A area	0.00081	0.00083	0.00039	0.00123	0.00193
concentration	2.18	2.12	1.5	2.59	5.14
	(76.99*)	(13.89*)	(1.3*)	(15.96*)	(31.47*)
Peak B area	0.00167	0.00172	0.00093	0.00193	0.00183
Concentration	4.51	4.37	3.53	4.05	4.88
	(62.83*)	(0*)	(11.85*)	(25.43*)	(3.29*)
Peak C area	0.00183	0.00163	0.00121	0.00123	0.00200
Concentration	4.95	4.16	4.61	4.69	5.32
	(35.04*)	(56.06*)	(3.5*)	(3.65*)	(6.50*)

* Average percentage reduction in concentrations between feed and permeate by DDM.

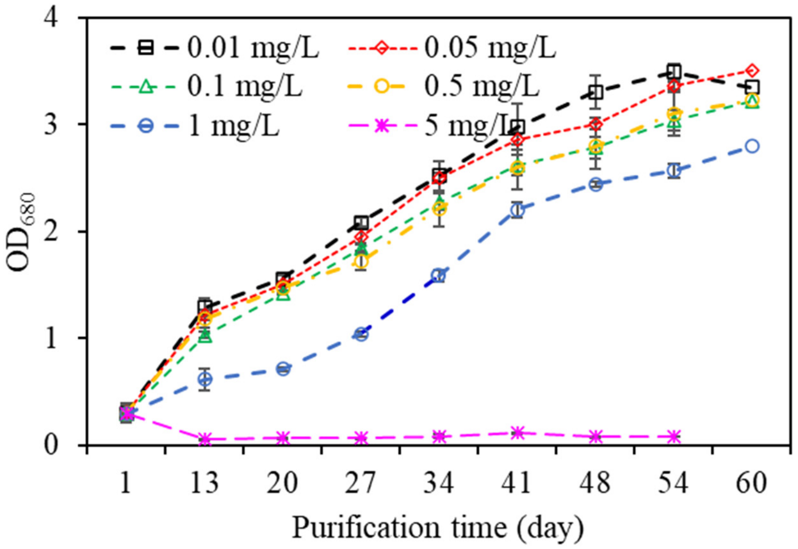

Figure S1. Growth of algae under various copper concentrations during aquaculture wastewater treatment.

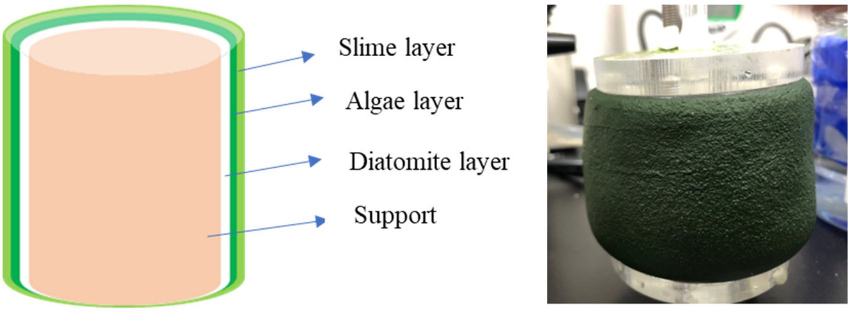

Figure S2. Schematic diagram of dynamic membrane structure.

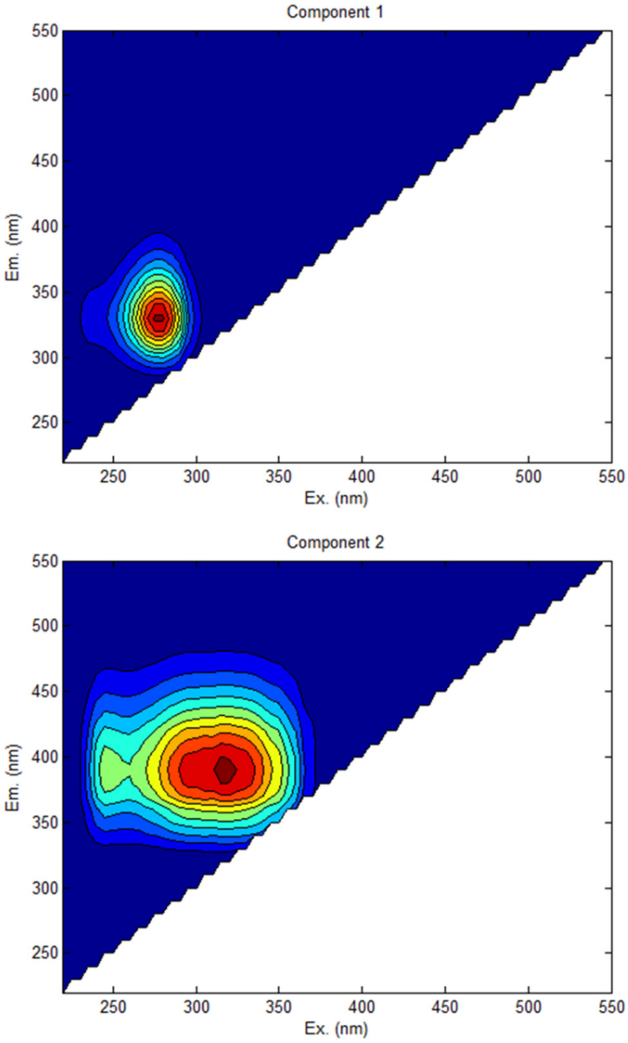

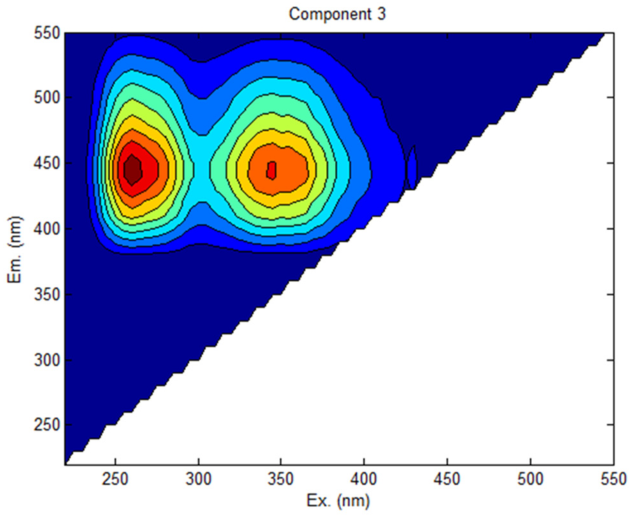

Figure S3. EEM spectra of EOM under various copper concentrations during purification process by EEM-PARAFAC.

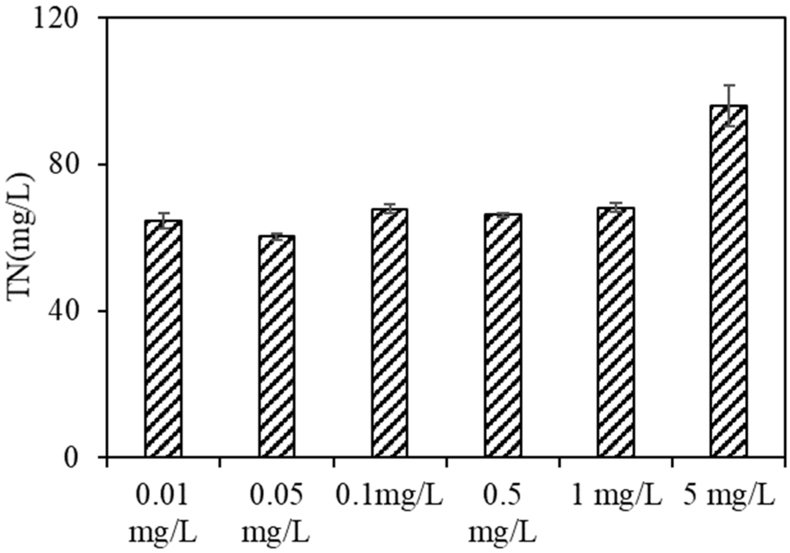

Figure S4. Total nitrogen content of algae under various copper concentrations after purification.

Fig. S1 shows the algal growth of *C. pyrenoidosa* during the purification process of aquaculture wastewater. Algae under low copper concentration (0.01 mg/L) had the highest growth rates, whereas the algal growth was gradually decreased with increasing with copper ions, especially when copper at high concentration of 5 mg/L, the algae were died after growing for a period of time, which suggested that copper ions in aquaculture wastewater have great influence on algal growth during the purification process, however, when high copper ions appeared, it was not conducive to be purified by *C. pyrenoidosa*. This result might be explained by the important coenzyme factor of copper ions during algal photosynthesis and respiration as well as the main component of photosynthetic transmission chain. Previous research suggested that many heavy metals, such as copper, zinc, and manganese, are necessary for the regeneration of algae cells at low concentrations, however, when they were at high concentrations, they were toxic.
